# Reversible Supramolecular Noncovalent Self-Assembly Determines the Optical Properties and the Formation of Melanin-like Nanoparticles

**DOI:** 10.1021/acs.jpclett.2c02239

**Published:** 2022-10-17

**Authors:** Alexandra Mavridi-Printezi, Arianna Menichetti, Lucia Ferrazzano, Marco Montalti

**Affiliations:** Dipartimento di Chimica “Giacomo Ciamician”, University of Bologna, Via Selmi 2, 40126Bologna, Italy

## Abstract

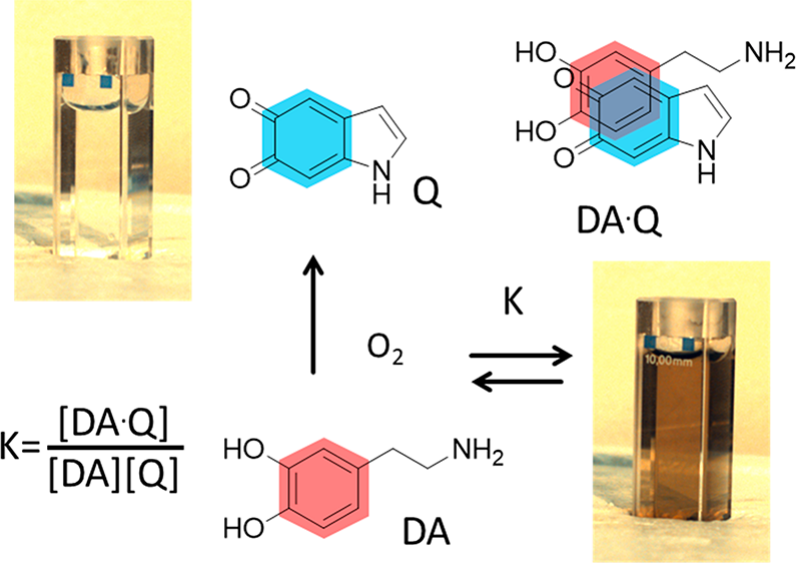

The role of noncovalent supramolecular self-assembly in the formation of melanin-like NP, as well as the nature of the electronic transition at the basis of their unique optical properties, is strongly debated. Here we demonstrate that, during the first stage of formation of synthetic melanin, polydopamine (PDA), a small fraction of the molecular precursor dopamine (DA) is oxidized to quinone (Q) and a simple supramolecular charge-transfer (CT) adduct is formed thanks to the electron donor and electron acceptor properties of DA and Q, respectively. This adduct, also detectable by HPLC-MS, presents the broad absorption band in the red-NIR region typical of melanin-like materials. Importantly, its disaggregation upon dilution can be easily detected since it leads to the disappearance of the CT band, indicating the reversibility of the process. Moreover, the stability constant *K* of the CT adduct could be obtained using a simple association model.

Melanin plays fundamental roles in Nature as pigment^1^ and photoprotecting^2^ and antioxidant agent^3,4^ in almost any kind of living organism.^5^ Materials that mimic the unique optical and electronic properties of melanin are finding, especially in virtue of their exceptional biocompatibility, technological application in fields of high economic and social impact including material science,^6^ environmental remediation,^7^ and in particular, nanomedicine.^8−12^ Considering this importance, the scientific community dedicated a great effort to understand the actual structure of melanin-like materials^13−17^ and the origin of their optical and electronic properties.^18−22^ Indirect evidence of a contribution of supramolecular self-assembly on the formation of complex melanin-like structure has been suggested by several authors,^23^ but a definitive demonstration of this noncovalent self-assembly process, and in particular of its reversibility, has not been reported up to now. In fact, pure supramolecular, noncovalent self-assembly is, at least from the thermodynamic point of view, expected to be a reversible, concentration-dependent process as observed for micellar systems, for molecular aggregates, or for electron donor–acceptor complexes^24,25^ that are at the basis of the formation of some rotaxanes and catenanes.^26^ Electron donor–acceptor complexes are particularly interesting in terms of optical properties since^27^ their assembly has been reported to produce relevant change in the light absorption properties because of the formation of new electronic transitions with charge-transfer (CT) character.^28^ It is fundamental to underline that CT electronic transitions are not localized on any of the molecular units constituting the system, but they arise from the interunit interaction and disappear as soon as the system disassembles in a reversible way.^24−27^ Here we will demonstrate, for the first time, that melanin-like materials behave, in the early stages of their formation, exactly in this way, showing reversible, concentration-dependent optical properties that reveal the supramolecular assembly and disassembly of molecular components.

In particular, we will focus on the first stages of the formation of polydopamine (PDA, the synthetic eumelanin analogue) NP synthesized starting from the molecular precursor dopamine (DA, Figure 1) upon exposure to atmospheric oxygen (see the Supporting Information for details). Since experimental parameters like pH, concentration, solvent composition, and kind of base are known to affect the process, they were carefully controlled in our experiments. These PDA NP have been reported to be formed through a complex series of oxidation and polymerization, leading to the formation of the characteristic broad band absorption spectrum of synthetic and natural melanin that extends from the UV to the NIR region, that confers to their water suspension a typical dark brown color (see the Supporting Information).^8,29^ After long exposure times of the reaction precursor to atmospheric oxygen (24 h or more), PDA NP can be indeed detected, in suspension, by dynamic light scattering (DLS, hydrodynamic diameter *d*_H_ = 140 nm) and optical transmission microscopy or observed by TEM or SEM (see the Supporting Information).

**Figure 1:**
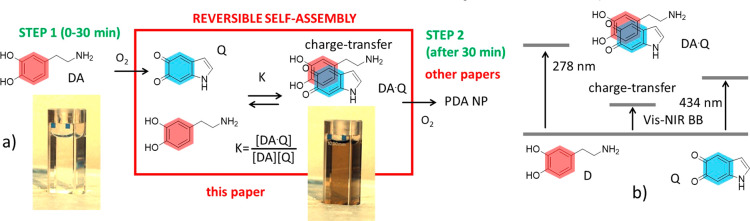
(a) Scheme of the self-assembly process occurring upon dopamine (DA) oxidation. Before the oxidation, the solution is not colored (cuvette on the left); after 30 min oxidation, the solution is brown (cuvette on the right) because of the formation of a CT adduct (with stability constant K) by the electron donor unit DA and the electron acceptor Q that is formed by partial oxidation of DA by atmospheric oxygen. (b) The supramolecular adduct presents a CT band, and differently from the components, it absorbs in the red-NIR region.

On the other hand, focusing on the first stages of the reaction that lead to the formation of PDA NP, we could observe that the appearance of the broad absorption band typical for melanin-like materials and hence of the dark-brown coloration (shown in Figure 2a) is not directly related to the PDA NP formation. In order to demonstrate this mismatch, we combined UV–vis absorption spectroscopy to DLS analysis, and dilution experiments as discussed below.

**Figure 2:**
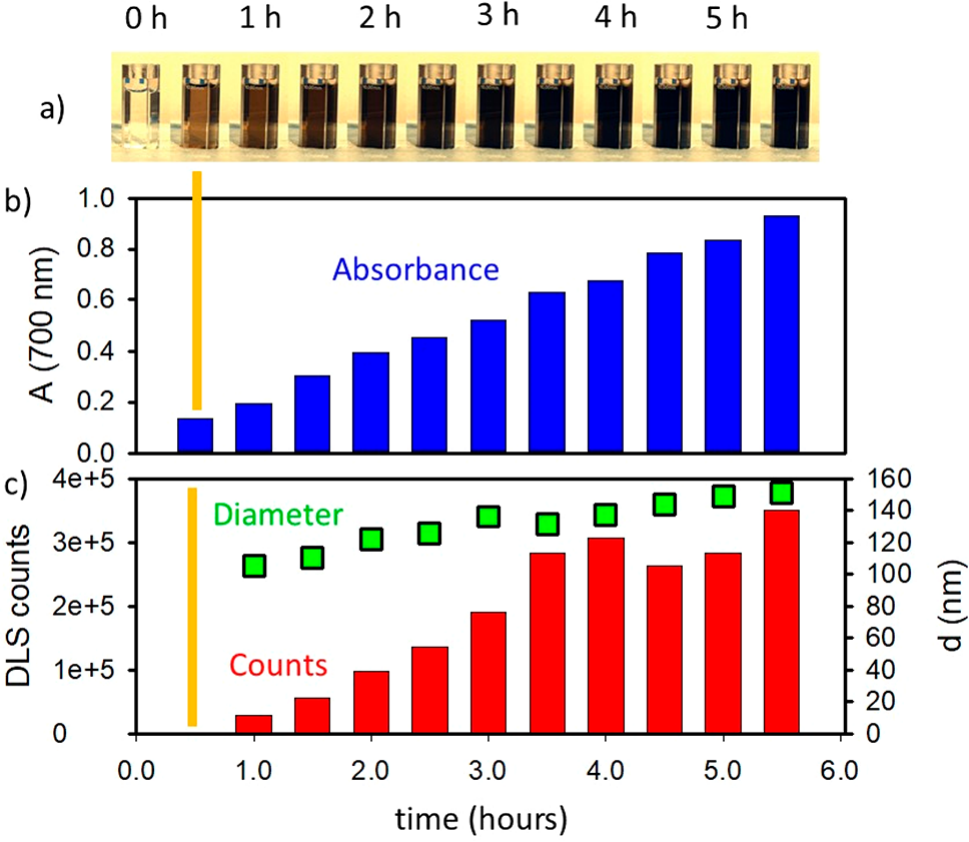
(a) Photographs of a solution of DA (15 mM) in EtOH/H_2_O 1:3 with NH_4_OH 0.1 M taken every 30 min after its preparation and exposure to atmospheric oxygen (the solution was continuously stirred). (b) Absorption of the solution shown in (a) at 700 nm (2 mm optical path) at different times. (c) Red bars. Light scattering intensity measured by DLS of the solution shown in (a) at different times. Green squares: hydrodynamic diameter of the NP formed in the solution shown in (a) as measured with DLS at different times.

In fact, the actual formation of PDA NP resulting from DA oxidation/polymerization,^30−32^ was investigated by DLS since this technique allows real time analysis of the reacting DA solution to detect NP formation. In particular, in Figure 2 we plotted the intensity of the scattering, which depends both on the concentration and on the size of the NP, and the measured hydrodynamic diameter.

Figure 2 clearly shows that the scattering intensity is negligible after 30 min of reaction, proving that no NP is formed in this time period. On the contrary, the scattering intensity becomes significant after 1 h of reaction and it increases linearly up to 4 h after it stops. This result indicates that the formation of PDA NP starts only 1 h after the beginning of the reaction and it ends after 4 h. Additionally, as shown in Figure 2, in this time interval the hydrodynamic diameter of the NP is quite constant around 130–140 nm.

These results confirm that the color change observed after 30 min of reaction (Figure 2b) is due to small, weakly light scattering species like molecular or supramolecular species. In order to demonstrate the actual presence of noncovalent self-assembled supramolecular structures acting as broad-band absorbing species after 30 min of reaction, we performed simple dilution experiments.

As discussed for other supramolecular systems,^27^ in the case of the formation of colored adducts resulting from noncovalent supramolecular self-assembly, disaggregation is expected upon decreasing concentration with a consequent disappearance of the CT band. Hence, in order to demonstrate the actual reversibility of the spectral change in the case of PDA and the occurrence of a noncovalent self-assembly process leading to species showing CT absorption, we compared the absorption spectrum of the DA solution during PDA formation at two difference concentrations for 4 h (taking into consideration the dilution and difference in optical path). In particular, the first concentration, *c*_0_, corresponds to the reacting DA solution without any dilution with an absorption spectrum *A*_0_ measured in a 2 mm optical path. The second concentration, *c*_100_, resulted from a 100-time dilution of the *c*_0_ solution, while its absorption spectrum, *A*_100_, was measured in a 50 mm optical path.

As a consequence, considering the difference in concentration and optical path, in the case of no concentration-dependent absorption, it would be expected *A*_0_ = 4*A*_100_ (see the Supporting Information for details). On the contrary, in the case of disaggregation upon dilution with concomitant disappearance of CT absorption, 4*A*_100_ is expected to be lower than *A*_0_ in the absorption range of the CT transition.

In order to clarify that, in Figure 3 the absorption spectra *A*_0_ at different reaction times are reported as continuous lines and compared to the corresponding 4*A*_100_, represented as dashed lines. Figure 3 clearly shows that, during the first 4 h of reaction, 4*A*_100_ is considerably lower than *A*_0_. This result is extremely important since it demonstrates that the typical absorption bands of PDA in the red-NIR region are strongly reduced in intensity upon dilution during the first hours of PDA formation indicating that (i) the PDA absorption bands in the red-NIR region result, at least in part, from the reversible noncovalent self-assembly of molecular components and that (ii) the absorption bands are due to CT transitions. In order to rule out that the change in the absorption spectrum was due to simple hyper/hypochromic effect depending on the substrate concentration, we compared the absorption spectra of DA at the two concentration *c*_0_ and *c*_100_, observing that they perfectly overlap showing no absorption in the vis–NIR region (see the Supporting Information).

**Figure 3:**
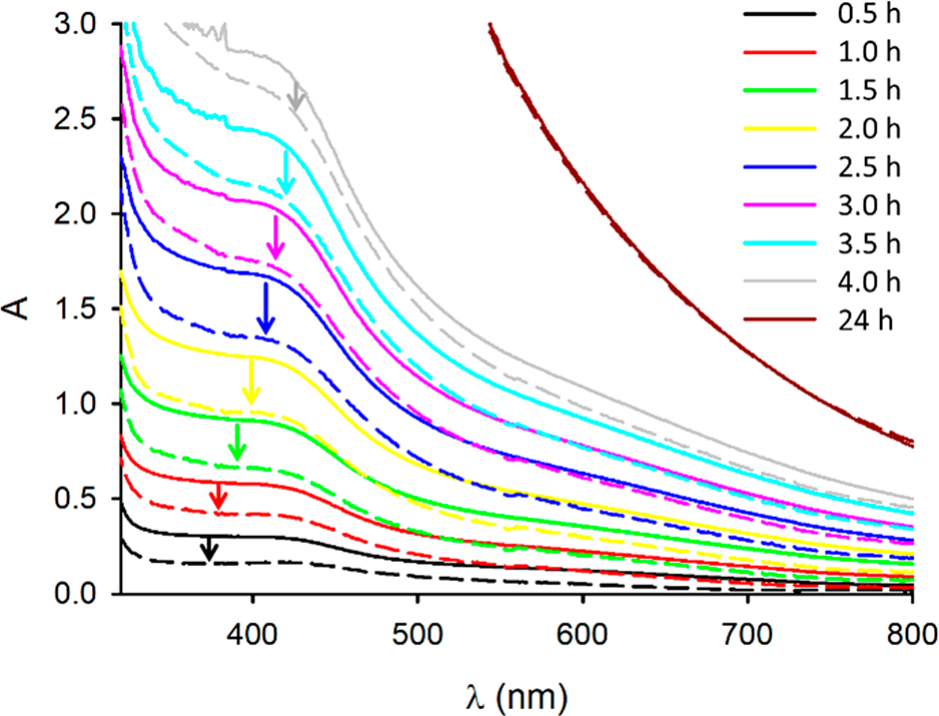
Continuous lines represent *A*_0_, which is the absorption spectra of a DA solution (15 mM) in EtOH/H_2_O 1:3 with NH_4_OH 0.1 M taken at different times after preparation and exposure to atmospheric oxygen. The solution was continuously stirred, optical path 2 mm. The same solutions were diluted 100 times, and the absorbance was measured on a 50 mm optical path. In order to allow a direct comparison of the absorbance, the trivial effects of simple dilution and different optical paths were corrected by multiplying the actual absorbance *A*_100_ by 4. Dashed lines represent 4*A*_100_.

Going more into detail, as discussed in the Supporting Information, the ratio between *A*_0_ and 4*A*_100_ can be used to quantify the fraction of aggregated molecules still present upon dilution. In particular, choosing 700 nm as a typical wavelength of the PDA absorption, the fraction of molecule  that is disaggregated after dilution will be given by
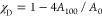
1

Therefore, the values of *A*_0_ and 4*A*_100_ at 700 nm, plotted in Figure 4, were used to calculate the fraction of disaggregated molecules upon 100 dilution according to eq 1. The results clearly show that, after 30 min of reaction, a fraction as high as 73% of the molecules undergoes disaggregation, while this fraction linearly decreases with reaction time.

**Figure 4:**
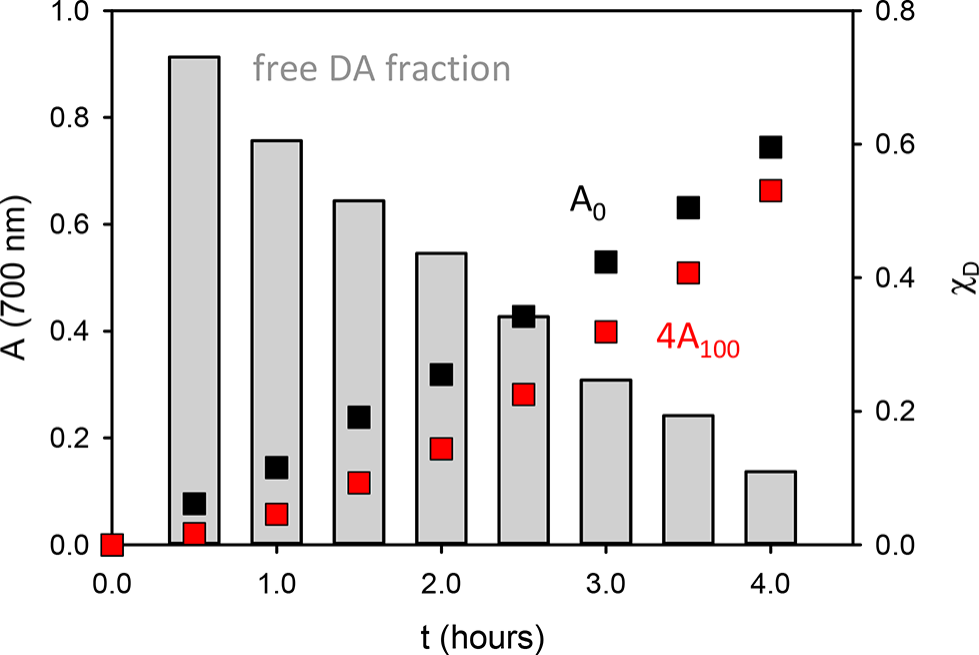
Black and red squares represent A_0_ and 4A_100_, shown in Figure 3 at 700 nm. These values were processed according to eq 1 to give the fraction of molecules undergoing disaggregation upon 100-time dilution (gray bars).

In particular, after 24 h, dilution produces no disaggregation and, as shown in Figure 3, *A*_0_ and 4*A*_100_ perfectly overlap, proving that an irreversible aggregation process occurs involving formation of covalent bonds occurred. Additionally, the absorbance *A*_0_ at 700 nm that increases linearly with time in the first hours of reaction does not change anymore.

Since this work is focused on the investigation of the reversible noncovalent self-assembly process occurring in the first stage of PDA formation, and since Figure 4 clearly shows that the contribution of this process is maximum after 30 min of reaction, we performed a deeper investigation of the reaction in the period of 30 min. In particular, in order to identify the supramolecular species formed in this stage, we used HPLC-MS. At the beginning of the reaction, only the protonated DA is detected with a *m*/*z* = 154.4, but after 30 min, of reaction a second species with *m*/*z* = 301.2 is detected. DA is known to undergo oxidation by oxygen, forming different quinones such as indole-5,6-quinone (Q, Figure 1). Interestingly, the species with *m*/*z* = 301.2 correspond to the protonated adduct formed by DA and indole-5,6-quinone (Q) schematized in Figure 1. This result demonstrates that, at the beginning of the PDA formation reaction, a small fraction of DA is oxidized by atmospheric oxygen to Q and then it assembles with the excess of DA to give a CT adduct. This process is driven by the electronic properties of DA and Q that, according to electrochemical measurement, are respectively a good electron donor and a good electron acceptor.^33,34^

The self-assembly process of DA and Q is schematized in Figure 1, and it can be modeled with very simple equations. In particular, in order to confirm the validity of the model schematized in Figure 1, we performed a new experiment where we measured the changes in the absorption spectrum of the DA solution after 30 min reaction upon different dilution (Figure 5), and not just upon 100-time dilution as in Figure 3.

**Figure 5:**
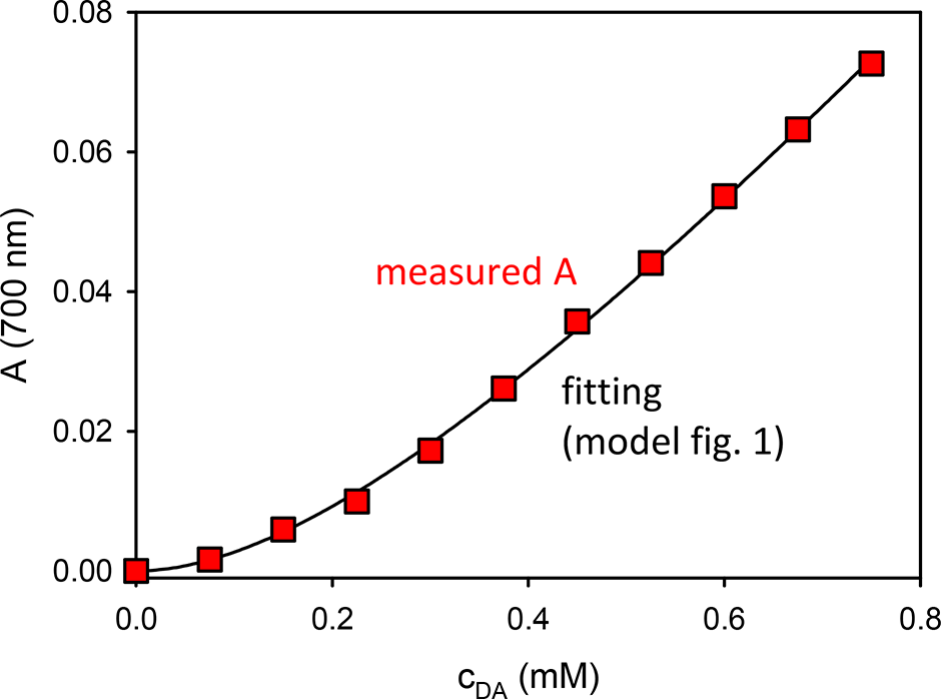
Red squares represent the absorbance at 700 nm of solutions obtained by different dilutions of a DA solution (15 mM) EtOH/H_2_O 1:3 with NH_4_OH 0.1 M that had been previously stirred for 30 min in the presence of atmospheric oxygen. On the abscissa, the concentration of DA at the different dilutions is reported. The plot was fitted according to eq 3 with the black curve, giving a *K* = 1952 M^–1^.

By considering the following simple association equilibrium (see the Supporting Information)
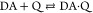
2with association constant *K*, a very simple dependency of the absorbance on the concentration of *D* is expected according to
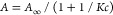
3where *c* is the concentration of DA, *A*_∞_ is a parameter describing the absorbance in the red-NIR region in the case of complete aggregation (see the Supporting Information), and *A* is the actual absorbance measured. As shown in Figure 5, the concentration-dependent absorption at 700 nm could be perfectly fitted with eq 3 giving K = 1952 M^–1^.

We would like to stress that FTIR, NMR, and XPS have been used in the past to investigate the chemical structure of melanin, giving important information. Nevertheless, these techniques require sample processing and are not fast enough for real-time investigation of the early stage of formation of PDA, which is the purpose of this work.

Moreover, even if a wide variety of species generated from DA have been detected in melanin-like nanoparticles after their complete formation and isolation, our results show that the early stage of the process involves the self-assembly of a single oxidized form of DA and DA itself.^23^ Our results are not in contrast with the previous works since they are compatible with a further evolution of the system to give a larger variety of species.

In conclusion, here we demonstrated that, in the first stage of the reaction of DA with atmospheric oxygen, a broad absorption band typical of melanin-like compounds is formed. This band has CT character and can be attributed to a simple supramolecular adduct composed by DA and its oxidized form, Q, that can be directly observed by LC–MS. The interaction between the electron donor species DA and the electron acceptor Q is purely noncovalent, and it is reversible upon dilution. Aggregation can be modeled with a simple one to one association stoichiometry giving a stability constant *K* = 1952 M^–1^. These important results demonstrate that (i) the broad bands in the red-NIR region typical of melanin have charge-transfer character and can be attribute to CT interactions between defined chromophoric units and (ii) the formation of melanin-like materials undergoes initial stages regulated by simple reversible supramolecular self-assembly. These results open a new perspective in the understanding of the complex process involved in the formation of melanin-like nanomaterials, species that are finding outstanding applications in important social and economic fields.

## Supporting Information Available

Experimental details, materials, methods, and synthesis and characterization; SEM and TEM images, MS, UV–vis, and absorption spectra; models for the experimental data fitting (PDF)
Change in color of a solution of DA during PDA formation (1 frame 10 min) (AVI)
Transmission image (100× magnification) of PDA NP in water (1 frame 30 ms) (AVI)

## Supplementary Material

jz2c02239_si_001.pdf

jz2c02239_si_002.avi

jz2c02239_si_003.avi

## References

[ref1] Battistella C.; McCallum N. C.; Gnanasekaran K.; Zhou X.; Caponetti V.; Montalti M.; Gianneschi N. C. Mimicking Natural Human Hair Pigmentation with Synthetic Melanin. ACS Cent. Sci. 2020, 6 (7), 1179–1188. 10.1021/acscentsci.0c00068. 32724852 PMC7379382

[ref2] Brenner M.; Hearing V. J. The protective role of melanin against UV damage in human skin. Photochem. Photobiol. 2008, 84 (3), 539–549. 10.1111/j.1751-1097.2007.00226.x. 18435612 PMC2671032

[ref3] Dias V.; Junn E.; Mouradian M. M. The role of oxidative stress in parkinson’s disease. J. Parkinsons Dis. 2013, 3 (4), 461–491. 10.3233/JPD-130230. 24252804 PMC4135313

[ref4] Panzella L.; Gentile G.; D’Errico G.; Della Vecchia N. F.; Errico M. E.; Napolitano A.; Carfagna C.; d’Ischia M. Atypical Structural and π-Electron Features of a Melanin Polymer That Lead to Superior Free-Radical-Scavenging Properties. Angew. Chem., Int. Ed. 2013, 52 (48), 12684–12687. 10.1002/anie.201305747. 24123614

[ref5] d’Ischia M.; Wakamatsu K.; Napolitano A.; Briganti S.; Garcia-Borron J.-C.; Kovacs D.; Meredith P.; Pezzella A.; Picardo M.; Sarna T.; Simon J. D.; Ito S. Melanins and melanogenesis: methods, standards, protocols. Pigm. Cell Melanoma R. 2013, 26 (5), 616–633. 10.1111/pcmr.12121. 23710556

[ref6] Lee H.; Dellatore S. M.; Miller W. M.; Messersmith P. B. Mussel-inspired surface chemistry for multifunctional coatings. Science 2007, 318 (5849), 426–430. 10.1126/science.1147241. 17947576 PMC2601629

[ref7] Wang Z.; Yang H. C.; He F.; Peng S.; Li Y.; Shao L.; Darling S. B. Mussel-Inspired Surface Engineering for Water-Remediation Materials. Matter 2019, 1 (1), 115–155. 10.1016/j.matt.2019.05.002.

[ref8] Liu Y.; Ai K.; Liu J.; Deng M.; He Y.; Lu L. Dopamine-melanin colloidal nanospheres: An efficient near-infrared photothermal therapeutic agent for in vivo cancer therapy. Adv. Mater. 2013, 25 (9), 1353–1359. 10.1002/adma.201204683. 23280690

[ref9] Cheng W.; Zeng X.; Chen H.; Li Z.; Zeng W.; Mei L.; Zhao Y. Versatile Polydopamine Platforms: Synthesis and Promising Applications for Surface Modification and Advanced Nanomedicine. ACS Nano 2019, 13 (8), 8537–8565. 10.1021/acsnano.9b04436. 31369230

[ref10] Carmignani A.; Battaglini M.; Sinibaldi E.; Marino A.; Vighetto V.; Cauda V.; Ciofani G. In Vitro and Ex Vivo Investigation of the Effects of Polydopamine Nanoparticle Size on Their Antioxidant and Photothermal Properties: Implications for Biomedical Applications. ACS Appl. Nano Mater. 2022, 5 (1), 1702–1713. 10.1021/acsanm.1c04536.

[ref11] Battaglini M.; Marino A.; Carmignani A.; Tapeinos C.; Cauda V.; Ancona A.; Garino N.; Vighetto V.; La Rosa G.; Sinibaldi E.; Ciofani G. Polydopamine Nanoparticles as an Organic and Biodegradable Multitasking Tool for Neuroprotection and Remote Neuronal Stimulation. ACS Appl. Mater. Interfaces 2020, 12 (32), 35782–35798. 10.1021/acsami.0c05497. 32693584 PMC8009471

[ref12] Wang T.; Niu K.; Ni S.; Zhang W.; Liu Z.; Zhang X. Hyaluronic Acid-Modified Gold-Polydopamine Complex Nanomedicine for Tumor-Targeting Drug Delivery and Chemo-Photothermal-Therapy Synergistic Therapy. ACS Sustain. Chem. Eng. 2022, 10 (4), 1585–1594. 10.1021/acssuschemeng.1c07231.

[ref13] Dreyer D. R.; Miller D. J.; Freeman B. D.; Paul D. R.; Bielawski C. W. Elucidating the structure of poly(dopamine). Langmuir 2012, 28 (15), 6428–6435. 10.1021/la204831b. 22475082

[ref14] Saiz-Poseu J.; Mancebo-Aracil J.; Nador F.; Busqué F.; Ruiz-Molina D. The Chemistry behind Catechol-Based Adhesion. Angew. Chem., Int. Ed. 2019, 58 (3), 696–714. 10.1002/anie.201801063. 29573319

[ref15] Della Vecchia N. F.; Avolio R.; Alfè M.; Errico M. E.; Napolitano A.; D’Ischia M. Building-block diversity in polydopamine underpins a multifunctional eumelanin-type platform tunable through a quinone control point. Adv. Funct. Mater. 2013, 23 (10), 1331–1340. 10.1002/adfm.201202127.

[ref16] D’Ischia M.; Napolitano A.; Ball V.; Chen C. T.; Buehler M. J. Polydopamine and eumelanin: From structure-property relationships to a unified tailoring strategy. Acc. Chem. Res. 2014, 47 (12), 3541–3550. 10.1021/ar500273y. 25340503

[ref17] Cao W.; Zhou X.; McCallum N. C.; Hu Z.; Ni Q. Z.; Kapoor U.; Heil C. M.; Cay K. S.; Zand T.; Mantanona A. J.; Jayaraman A.; Dhinojwala A.; Deheyn D. D.; Shawkey M. D.; Burkart M. D.; Rinehart J. D.; Gianneschi N. C. Unraveling the Structure and Function of Melanin through Synthesis. J. Am. Chem. Soc. 2021, 143 (7), 2622–2637. 10.1021/jacs.0c12322. 33560127

[ref18] Grieco C.; Kohl F. R.; Hanes A. T.; Kohler B. Probing the heterogeneous structure of eumelanin using ultrafast vibrational fingerprinting. Nat. Commun. 2020, 11 (1), 4569 10.1038/s41467-020-18393-w. 32917892 PMC7486937

[ref19] Kohl F. R.; Grieco C.; Kohler B. Ultrafast spectral hole burning reveals the distinct chromophores in eumelanin and their common photoresponse. Chem. Sci. 2020, 11 (5), 1248–1259. 10.1039/C9SC04527A. PMC8148383 34123249

[ref20] Ju K.-Y.; Fischer M. C.; Warren W. S. Understanding the Role of Aggregation in the Broad Absorption Bands of Eumelanin. ACS Nano 2018, 12 (12), 12050–12061. 10.1021/acsnano.8b04905. 30500158

[ref21] d’Ischia M.; Napolitano A.; Pezzella A.; Meredith P.; Sarna T. Chemical and Structural Diversity in Eumelanins: Unexplored Bio-Optoelectronic Materials. Angew. Chem., Int. Ed. 2009, 48 (22), 3914–3921. 10.1002/anie.200803786. PMC2799031 19294706

[ref22] d’Ischia M.; Napolitano A.; Ball V.; Chen C.-T.; Buehler M. J. Polydopamine and Eumelanin: From Structure–Property Relationships to a Unified Tailoring Strategy. Acc. Chem. Res. 2014, 47 (12), 3541–3550. 10.1021/ar500273y. 25340503

[ref23] Hong S.; Na Y. S.; Choi S.; Song I. T.; Kim W. Y.; Lee H. Non-covalent self-assembly and covalent polymerization co-contribute to polydopamine formation. Adv. Funct. Mater. 2012, 22 (22), 4711–4717. 10.1002/adfm.201201156.

[ref24] Crisenza G. E. M.; Mazzarella D.; Melchiorre P. Synthetic Methods Driven by the Photoactivity of Electron Donor–Acceptor Complexes. J. Am. Chem. Soc. 2020, 142 (12), 5461–5476. 10.1021/jacs.0c01416. 32134647 PMC7099579

[ref25] Foster R. Electron donor-acceptor complexes. J. Phys. Chem. 1980, 84 (17), 2135–2141. 10.1021/j100454a006.

[ref26] Balzani V.; Credi A.; Raymo F. M.; Stoddart J. F. Artificial molecular machines. Angew. Chem., Int. Ed. 2000, 39 (19), 3348–3391. 10.1002/1521-3773(20001002)39:19<3348::AID-ANIE3348>3.0.CO;2-X. 11091368

[ref27] Credi A.; Montalti M.; Balzani V.; Langford S. J.; Raymo F. M.; Stoddart J. F. Simple molecular-level machines. Interchange between different threads in pseudorotaxanes. New J. Chem. 1998, 22 (10), 1061–1065. 10.1039/a804787a.

[ref28] Asakawa M.; Ashton P. R.; Balzani V.; Credi A.; Hamers C.; Mattersteig G.; Montalti M.; Shipway A. N.; Spencer N.; Stoddart J. F.; Tolley M. S.; Venturi M.; White A. J. P.; Williams D. J. A Chemically and Electrochemically Switchable [2]Catenane Incorporating a Tetrathiafulvalene Unit. Angew. Chem., Int. Ed. 1998, 37 (3), 333–337. 10.1002/(SICI)1521-3773(19980216)37:3<333::AID-ANIE333>3.0.CO;2-P. 29711270

[ref29] Huang Y.; Li Y.; Hu Z.; Yue X.; Proetto M. T.; Jones Y.; Gianneschi N. C. Mimicking Melanosomes: Polydopamine Nanoparticles as Artificial Microparasols. ACS Cent. Sci. 2017, 3 (6), 564–569. 10.1021/acscentsci.6b00230. 28691067 PMC5492417

[ref30] Delparastan P.; Malollari K. G.; Lee H.; Messersmith P. B. Direct Evidence for the Polymeric Nature of Polydopamine. Angew. Chem., Int. Ed. 2019, 58 (4), 1077–1082. 10.1002/anie.201811763. PMC6424361 30485624

[ref31] Lee K.; Park M.; Malollari K. G.; Shin J.; Winkler S. M.; Zheng Y.; Park J. H.; Grigoropoulos C. P.; Messersmith P. B. Laser-induced graphitization of polydopamine leads to enhanced mechanical performance while preserving multifunctionality. Nat. Commun. 2020, 11 (1), 4848 10.1038/s41467-020-18654-8. 32973166 PMC7515926

[ref32] Ryu J. H.; Messersmith P. B.; Lee H. Polydopamine Surface Chemistry: A Decade of Discovery. ACS Appl. Mater. Interfaces 2018, 10 (9), 7523–7540. 10.1021/acsami.7b19865. 29465221 PMC6320233

[ref33] Raymo F. M.; Cejas M. A. Supramolecular Association of Dopamine with Immobilized Fluorescent Probes. Org. Lett. 2002, 4 (19), 3183–3185. 10.1021/ol026412p. 12227744

[ref34] Sindelar V.; Cejas M. A.; Raymo F. M.; Chen W.; Parker S. E.; Kaifer A. E. Supramolecular assembly of 2,7-dimethyldiazapyrenium and cucurbit[8]uril: A new fluorescent host for detection of catechol and dopamine. Chem.—Eur. J. 2005, 11, 7054–7059. 10.1002/chem.200500917. 16175642

