# Supplementary material for: Reversible Supramolecular Noncovalent Self-Assembly Determines the Optical Properties and the Formation of Melanin-like Nanoparticles
Source: J Phys Chem Lett. 2022 Oct 17;13(42):9829–33. doi: 10.1021/acs.jpclett.2c02239 (PMC9620075; doi:10.1021/acs.jpclett.2c02239)
Supplement: Supplementary file 1 — jz2c02239_si_001.pdf [file jz2c02239_si_001.pdf]

Supporting Information

Reversible Supramolecular Non-Covalent Self-Assembly Determines the Optical Properties and the Formation of Melanin-Like Nanoparticles.

Alexandra Mavridi-Printezi, Arianna Menichetti, Lucia Ferrazzano and Marco Montalti*

Dipartimento di Chimica "Giacomo Ciamician", University of Bologna, Via Selmi 2, 40126 Bologna (Italy)
E-mail: marco.montalti2@unibo.it.

1. Materials and Methods

1.1 Materials

All reagents, solvents and chemicals were purchased from Sigma-Aldrich and used without modification, unless otherwise stated.

1.2 UV-Vis Spectroscopy

The experiments were carried out in air-equilibrated solutions at 25 °C. UV–Vis absorption spectra were recorded with a Perkin-Elmer Lambda 45 spectrophotometer using quartz cells with different path lengths that were 1.0 cm, 2mm and 50 mm.

1.3 HPLC-MS

The Mass spectra were received using a 1260 Infinity II LC System with a Luna Phenomenex C18 column. As mobile phase, mixture of H_2O: ACN both acidified by 0.1% TFA was used. The flux was 0.4 ml/min with gradient of solvents in time reported in Table S1. The parameters for the mass acquisition were the following ion source: API-ES, mode: Positive scan, fragmentor: 30 eV, Gain: 1, Mass range: 50-3000 m/z, Drying gas flow: 8 l/min, Nebulizer pressure: 30 psig, Drying gas temperature: 350 °C, Capillary voltage: 4000 V.

Time (min)	Water (%)	Organic (%)
**0**	70	30
**8**	20	80
**22**	20	80
**24**	10	90
**26**	10	90
**28**	70	30

Table S1. Gradient of solvents in time during the HPLC-MS operation

1.4 Optical Microscopy

The nanoparticle videos in transmission mode were obtained using an Olympus IX 71 inverted microscope equipped with an ultrasensitive EMCCD Princeton PhotonMAX camera (512x512 pixel, pixel size 16x16 μm^2) camera. The samples were placed within two glass slides separated by a 0.1 mm spacer. Glass slides were Aptaca 26x76 microscope slides with thickness 1.00/1.20 mm and the Aptaca 24x50 glass slides with thickness 0.13/0.17 mm. In particular, the images were collected by a 100X oil immersion objective (Olympus UPLFLN 100X). For the videos, the images were collected in time lapse mode at a rate of one image every 30 ms for a total time of 15 s.

1.5 Video acquisition of Polydopamine (PDA) colour change during synthesis

PDA synthesis was monitored in time in a synthesis carried out in a quartz cuvette of 1.0 cm path length. The colour formation was recorded with a Basler acA5472-17uc colour camera with a Sony IMX183 CMOS sensor. The software for the acquisition was the IC-Capture receiving a frame every 5 minutes. The images were afterwards elaborated using ImageJ software.

1.6 Dynamic Light Scattering (DLS)

DLS measurements were performed with Zetasizer Nano ZS Malvern Panalytical using PMMA semi-micro cuvettes (BRAND).

1.7 Transmission Electron Microscopy (TEM)

TEM images were received using a Philips TEM CM 100 electron microscope at an accelerating voltage of 80 kV. For the acquisition, the samples were deposited on Formvar on 400 mesh Cu grids supplied by TED PELLA INC.

1.8 Scanning Electron Microscopy (SEM)

Scanning electron microscopy was performed using a Field Emission Scanning Electronic Microscope Mira3 by Tescan. In particular in column secondary electron detector was chosen in order to obtain higher performance. The view field was 1.04μm and the voltage was 15.0 kV with magnification (MAG) 267 kx and working distance (WD) of 7.01 mm. Other parameters used were view field 2.08 μm, voltage 4.0kV with MAG 133kx and WD 4.04 mm.

2. Synthesis and Characterization of PDA

2.1 Synthesis of Polydopamine (PDA)

Polydopamine (PDA) was prepared by the spontaneous oxidation of dopamine hydrochloride in aerobic and alkaline conditions.[1] Initially, 40 mL of ethanol and 90 ml of deionized water were mixed in a 250 mL round bottom flask. In the flask were added 2 mL of 28-30% NH_4OH and the mixture was stirred vigorously for 10 min. In the next step, a dopamine hydrochloride solution was prepared by solubilizing 400 mg of dopamine hydrochloride in 10 mL of deionized water. The dopamine solution was injected fast into the flask under

vigorous stirring. An immediate colour change was observed which evolved in time from light yellow to dark brown. The reaction was carried out for 24hours under vigorous stirring. After 24 hours, the nanoparticles (NPs) were retrieved by centrifugation at 14000 rpm for 20minutes. Then, they were washed with deionized water three times at 10.000rpm and finally redispersed in water.

2.2 DLS of PDA and NPs

For the DLS, PDA directly after the synthesis was diluted 500 times in deionized water while a double dilution was realised for the NPs. PDA and NPs water-dispersions possessed nanoparticles of around 120 nm and a very narrow size distribution indicated by the low Polydispersity Index (PDI). (Table S2).

Sample	Z-Average	PDI	Attenuator
**PDA**	122.1	0.003	9
**NPs**	127.2	0.024	8

Table S2. DLS results of PDA and water- dispersion of NPs after their purification

2.3 SEM and TEM of PDA NPs

TEM and SEM images of PDA NPs were retrieved after their purification and redispersion in water. Both SEM (Figure S1) and TEM (Figure S2) images revealed the presence of spherical particles with diameter around 100nm.

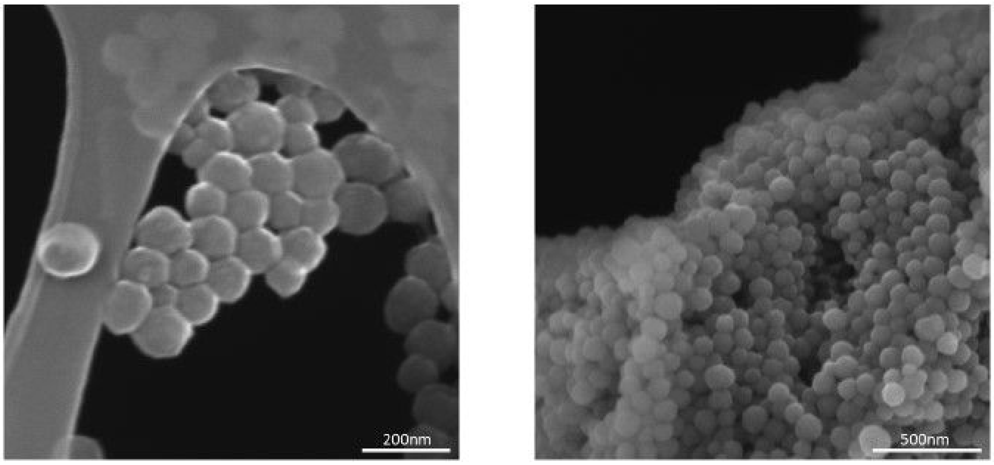

Figure S1. SEM images of PDA NPs.

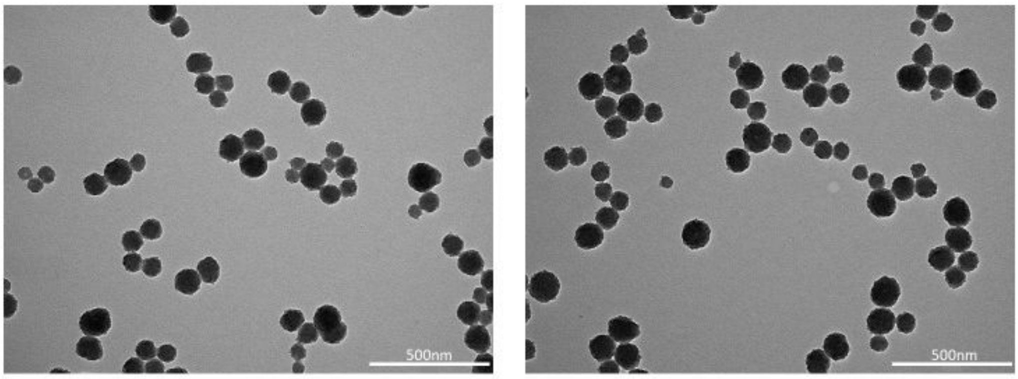

Figure S2. TEM images of PDA NPs.

HPLC-MS

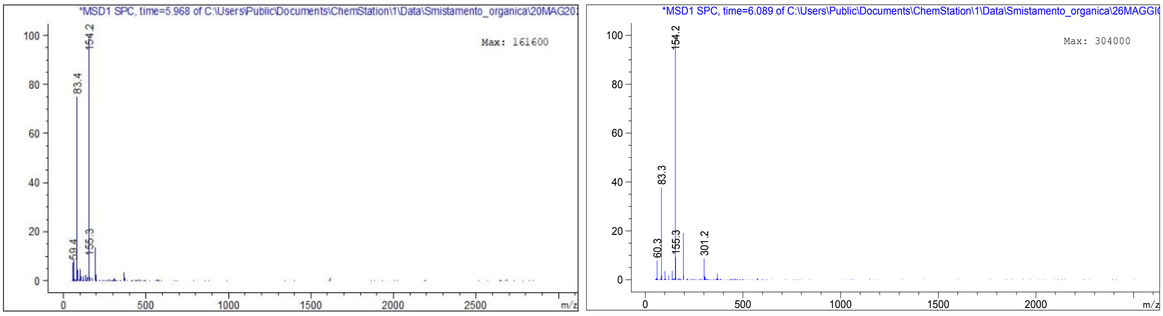

Figure S3. Left: Mass spectrum recorder at the beginning of the reaction and showing the presence of DA (m/z=154.2). Right: Mass spectrum recorded after 30 min reaction and showing an additional peak corresponding to DA_Q (m/z=301.2).

2.4 UV-Vis Absorbance Spectroscopy

For the acquisition of the UV-Vis absorbance spectra, two different concentrations were exploited in order to investigate the formation of the electron donor-acceptor complex. In particular, 0.6 ml of sample were received every 30 minutes and placed in a 2 mm optical path cuvette without any treatment. At the same time, a 100 dilution of the reaction batch in the reaction mixture (water/ethanol/ammonia) was realized after degassing a 50 mm cuvette with Nitrogen to avoid further oxidation of the reaction mixture upon dilution.

2.6 Association Model and Fitting

Experimental data were fitted according to the simple association model:

$$DA + Q \rightleftarrows DA\_Q$$

$$k = \frac{[DA\_Q]}{[DA][Q]}$$

where *k* is the association constant (or stability constant) of the electron donor-acceptor complex DA_Q.

After 30 minutes of reaction, only a small fraction of DA is oxidized to Q hence, we can assume that [DA]=c where c is the initial concentration of DA.

The Total concentration of Q is indicated as fQ (where f<<1). Consequently [DQ]=x, [Q]=fQ-x and:

$$k = \frac{x}{(c-x)(fc-x)}$$

Being x<<c:

$$k = \frac{x}{c(fc-x)}$$

$$1 + \frac{1}{kc} = \frac{fc}{x}$$

In the red-NIR region neither DA or Q absorb light so at 700 nm:

$$A = x\varepsilon$$

Where ε is the molar absorption coefficient of DA_Q.

If the concentration c is high enough, the aggregation is complete and according to the model:

$$A_\infty = fc\varepsilon$$

As a consequence: $\frac{A}{A_\infty} = \frac{x}{fc}$

According to the previous, the following equation is obtained, which is Eq. 3 in the main text:

$$A = \frac{A_\infty}{1 + \frac{1}{kc}}$$

Additionally, at high concentration c_0 the produced Q is totally associated to the excess of DA and hence:

$$[DA\_Q]_0 = \frac{A_0}{\varepsilon}$$

Upon dilution, and therefor at a concentration c_{100}:

$$[DA\_Q]_{100} = \frac{4A_{100}}{\varepsilon}$$

Hence, the fraction of complex that is still associated after dilution is:

$$\frac{[DA\_Q]_{100}}{[DA\_Q]_0} = \frac{4A_{100}}{A_0}$$

and the fraction of complex that disassemble is:

which is Eq. 1 in the main text.

2.5 Absorption spectra of DA at different concentration.

In order to rule out that the change in the absorption spectrum was due to simple hyper/hypochromic effect depending on the substrate concentration we compared the absorption spectra of DA at the two concentrations c_0=15 mM (in a 2 mm cuvette) and c_{100}=0.15 mM (in a 40 mm cuvette). The spectra are shown in figure S4, the spectrum at c_{100} was multiplied by 5 in order to allow the comparison (considering the difference in optical path). The figure shows that the two spectra overlap perfectly and that they show no absorption in the Vis-NIR region.

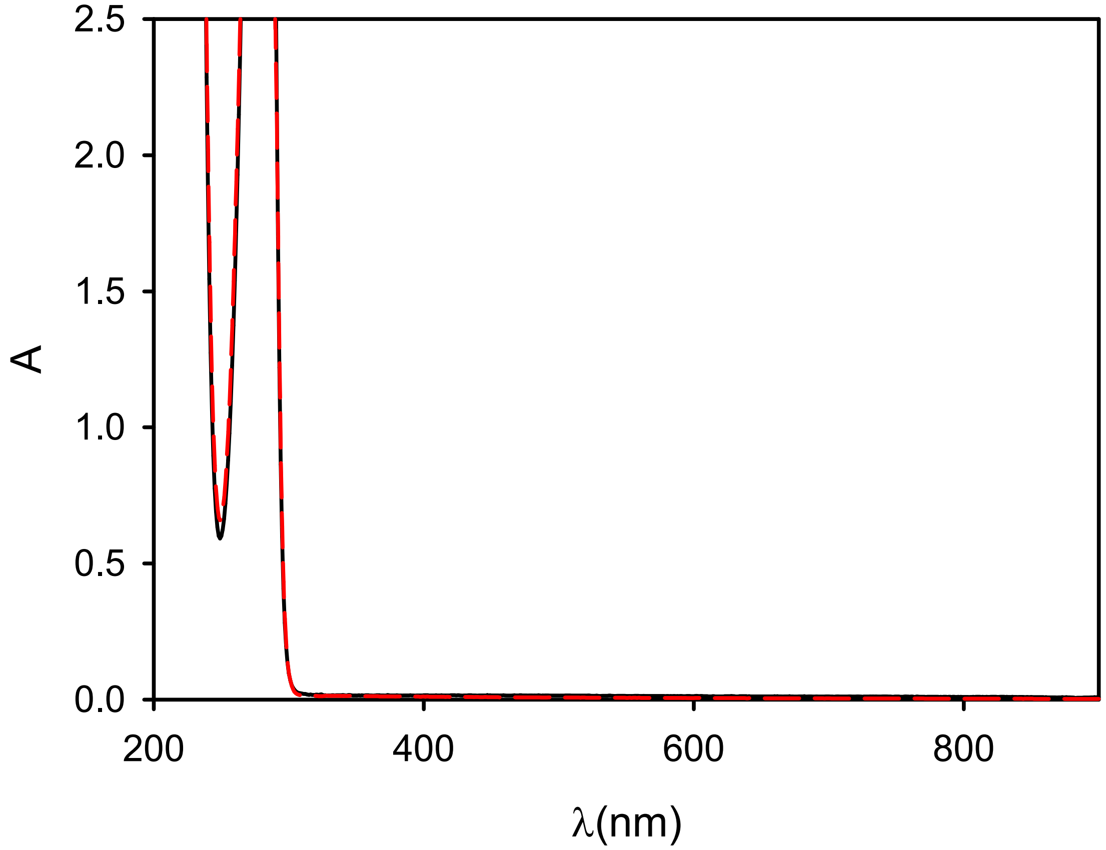

Figure S4. Absorption spectrum of DA at concentration c_0=15 mM (black continuous line, 2 mm cuvette) and at concentration c_{100}=0.15 mM (red dashed line, 40 mm cuvette, multiplied by 5 for comparison)

3. Bibliography

[1] Y. Huang, Y. Li, Z. Hu, X. Yue, M. T. Proetto, Y. Jones, N. C. Gianneschi, *ACS Cent. Sci.* **2017**, *3*, 564–569.
